# Study of pH Role in the Green Synthesis of Gold Nanoparticles: From Reduction to Size and Shape Control

**DOI:** 10.3390/ma19132780

**Published:** 2026-06-30

**Authors:** Oksana Velgosova, Maksym Lisnichuk, Jesús Hernández-Saz

**Affiliations:** 1 Faculty of Mechanical Engineering, Technical University of Košice, Letná 9/A, 04200 Kosice, Slovakia; 2 Faculty of Science, Institute of Physics, Pavol Jozef Šafarik University in Kosice, Park Angelinum 9, 04001 Kosice, Slovakia; maksym.lisnichuk@upjs.sk; 3 Institute of Materials Research of the Slovak Academy of Sciences, Watsonova 47, 04001 Kosice, Slovakia; 4 Department of Engineering and Materials Science and Transportation, University of Seville, Camino de los Descubrimientos s/n, 41092 Seville, Spain; jhernandez32@us.es

**Keywords:** gold, nanoparticles, green synthesis, shape, pH, extract concentration

## Abstract

This study investigates the influence of pH and extract concentration on the green synthesis of gold nanoparticles (AuNPs) using *Camellia sinensis* extract as a reducing and stabilizing agent. Synthesis was performed at pH 1–12 using undiluted and diluted extracts (2:5 and 1:5). The colloids were characterized by UV–Vis spectroscopy, TEM, SAED, and elemental mapping. Both parameters significantly affected nanoparticle formation, morphology, and optical properties. At extreme pH values and low extract concentrations, AuNP formation was suppressed. The pH range of 3–6 produced diverse morphologies: spherical (~18 nm), triangular (~21 nm), rod-shaped (~25 nm), and anisotropic flower-like structures (~53 nm). At higher pH values, smaller, more uniform spherical nanoparticles formed, including red (~18 nm) and yellow colloids (~6 nm). These changes were reflected in distinct UV–Vis spectra and color variations. Toxicity tests using *Chlorella kessleri* revealed no inhibition of algal growth. In contrast, selected AuNPs colloids reduced germination and root growth of Sinapis alba, with germination falling below 50% for spherical AuNPs smaller than 18 nm and root length decreasing by more than 60% compared to the positive control. Under the tested conditions, controlled adjustment of pH and extract concentration allows tuning of AuNP properties through a simple green synthesis approach.

## 1. Introduction

Nanotechnology is currently one of the most rapidly advancing fields of science, with gold nanoparticles (AuNPs) occupying a prominent position due to their unique optical, catalytic, and biological properties [1,2]. They are widely applied in areas such as biomedicine, diagnostics, targeted drug delivery [3], and environmental technologies [4,5,6]. Conventional physical and chemical methods for nanoparticle synthesis frequently involve hazardous chemicals, elevated temperatures, and energy-intensive processing, raising concerns regarding their environmental sustainability and large-scale applicability [7,8]. For this reason, biological (so-called “green”) synthesis has gained significant attention in recent years, as it utilizes plant extracts, microorganisms, or biomolecules as reducing and stabilizing agents [8,9].

One of the key factors influencing the biological synthesis of AuNPs is the pH of the reaction medium. The pH value significantly affects not only the reduction process of gold ions (Au^3+^ → Au^0^) but also the activity and ionization state of biomolecules present in biological extracts, such as phenols, flavonoids, and proteins. These molecules play a crucial role in the nucleation, growth, and stabilization of nanoparticles in solution [10,11]. Changes in pH can therefore lead to substantial differences in reaction kinetics and in the mechanisms of nanoparticle formation.

Several studies have shown that pH strongly influences the size, shape, and dispersity of gold nanoparticles [12,13,14,15]. At higher pH values (typically neutral to mildly alkaline), gold ion reduction occurs more rapidly, yielding smaller, often more uniform spherical nanoparticles [15]. In contrast, acidic environments slow nucleation, producing larger and more morphologically diverse structures [15,16].

However, it should be emphasized that these observations represent generalized trends rather than universal rules. Reported results are often system-dependent and may vary significantly depending on the nature and composition of the biological reducing agent. Therefore, the influence of pH cannot be considered independently of the specific extract used. Each biological reducing system exhibits unique chemical characteristics, and its behavior must be evaluated individually. Consequently, systematic investigation of synthesis conditions for a given reductant is essential, as broadly applicable conclusions across different plant extracts or biomolecular systems cannot be reliably established.

Alongside pH, the composition and concentration of plant extracts are critical factors in the green synthesis of AuNPs. These extracts contain a complex mixture of bioactive compounds, including polyphenols, alkaloids, terpenoids, and reducing sugars, which serve as both reducing and capping agents [17]. The amount of these compounds directly affects the number of electrons available to reduce gold ions and the stabilization of the resulting nanoparticles. Higher extract concentrations generally lead to faster nucleation because of the greater number of reducing agents, which can produce smaller nanoparticles, while lower concentrations may cause incomplete reduction or particle clumping. Therefore, controlling the extract concentration is vital for consistent synthesis and for customizing nanoparticle properties.

Control over nanoparticle shape and size is of key importance, as these parameters directly determine the optical response and resulting color of the colloidal system. Such tunable optical properties are highly desirable in applications including the coloration of polymers, glass, and coatings, where nanoparticle-based coloration offers superior stability compared to conventional pigments, as it does not fade over time. In addition, the color variations of AuNPs enable their use in colorimetric sensing for the detection of metal ions, biomolecules, and pathogens, as well as in biomedical imaging, photothermal therapy, and analytical techniques such as UV–Vis spectroscopy. Consequently, the ability to reproducibly prepare nanoparticles with defined morphology is essential for tailoring their optical properties for specific applications. Although a wide range of colors and morphologies can be achieved using conventional chemical synthesis methods [18], the present results demonstrate that similar control can also be attained via green synthesis. By simply adjusting pH and the concentration of the biological reducing agent, it is possible to tailor the optical properties of AuNPs, highlighting the potential of environmentally friendly approaches for preparing functional nanomaterials with tunable color.

Although the green synthesis of AuNP using plant extracts has been widely investigated, the combined effect of pH and extract concentration over a broad pH range has not yet been systematically evaluated. Limited attention has been paid to the relationship between these synthesis parameters and the resulting nanoparticle morphology, optical properties, and biological effects. Therefore, this study investigates the influence of pH on the biological synthesis of gold nanoparticles and their physicochemical properties while also evaluating the role of plant extract concentration as a key parameter affecting the reduction process and nanoparticle characteristics. The synthesis was systematically examined over a pH range of 1–12 using three extract concentrations. Based on the experimental results, the main factors governing AuNPs formation are discussed, and suitable conditions for preparing nanoparticles with selected properties are identified.

## 2. Materials and Methods

### 2.1. Materials

Hydrogen tetrachloroaurate (HAuCl_4_, 1000 mg/L standard solution, Merck, Germany) was used as the gold precursor. Green tea (*Camellia sinensis*) extract served as the reducing and stabilizing agent. Sodium hydroxide (NaOH, analytical grade, Centralchem, Bratislava, Slovakia) was used for pH adjustment. All solutions were prepared and diluted using deionized water.

### 2.2. Solution Preparation and Synthesis of AuNPs

#### 2.2.1. Plant Extract Preparation

Dry green tea leaves were added to 250 mL of distilled water. The mixture was heated to 75 °C under continuous stirring at 500 rpm. After reaching the target temperature, the mixture was stirred at 75 °C for an additional 10 min to ensure efficient extraction. The suspension was then filtered through filter paper and centrifuged at 9000 rpm for 15 min to remove residual solid particles. The clear extract was either used immediately for AuNP synthesis or stored at 4 °C and used within five days of preparation.

#### 2.2.2. Synthesis of Nanoparticles

A gold ionic solution concentration of 50 mg/L was used as a metal precursor for the synthesis of Au nanoparticles. Briefly, 50 mL of the metal precursor solution was heated to 75 °C. After reaching the target temperature, 5 mL of the plant extract was added under continuous stirring. Subsequently, the resulting colloidal suspension was allowed to cool to room temperature, followed by UV–Vis spectrophotometric analysis and pH measurement. Following synthesis, the AuNP suspensions were used directly for characterization and biological testing without any separation, washing, or purification step.

To evaluate the influence of pH on the synthesis of AuNPs, the metal precursor pH was adjusted before the synthesis. The pH was modified to values from 1 to 12 using 10 wt. % NaOH solution. In selected experiments, the concentration of the plant extract was also varied to further influence nanoparticle formation.

### 2.3. Methods

The optical properties of the synthesized AuNPs were evaluated by UV–Vis spectroscopy using a UNICAM UV4 spectrophotometer (Thermo Scientific, USA). Detailed morphological features and particle size were examined by transmission electron microscopy (TEM) (JEOL Ltd., Tokyo, Japan) operated at an accelerating voltage of 200 kV. For TEM analysis, a drop of the colloidal suspension was deposited onto a carbon-coated copper grid and dried at room temperature. Before analysis, the samples were coated with a thin carbon layer using a vacuum carbon evaporator (JEOL JEE 4C, Ionization Vacuum Gauge, Tokyo, Japan) to improve their stability under the electron beam. The elemental composition distribution of the nanoparticles was mapped using Energy-Dispersive X-ray Spectroscopy (EDX) (Thermo Fisher Scientific, Waltham, MA, USA) with four integrated Bruker Silicon Drift Detectors (SDD) (Bruker, Berlin, Germany). The particle size distribution was determined by digital image analysis using ImageJ software (version 1.54G; National Institutes of Health, Bethesda, MD, USA). The pH values of the reaction systems were monitored using an Orion Star A214 pH meter (Thermo Scientific, Bremen, Germany).

### 2.4. Toxicity Tests

The toxicity of the synthesized gold nanoparticles was assessed using two biological test systems. The first assay was performed with the green microalga *Chlorella kessleri* using a disk diffusion method. Sterile disks containing the tested AuNP samples were applied onto agar plates inoculated with the algal culture. The plates were subsequently incubated under controlled conditions. Toxicity was evaluated based on the presence or absence of inhibition zones around the disks, reflecting the effect of the tested samples on algal growth.

The second test focused on the root growth inhibition of the higher plant *Sinapis alba*. Seeds were exposed to the test samples under controlled conditions, and after a defined cultivation period (3 days), seed germination and root elongation were measured. Toxicity was assessed as the number of germinated seeds (%), and root length was measured and compared to the control. This test provides information on the phytotoxic potential of the samples and allows assessment of their influence on early plant development. Together, these two assays enabled the evaluation of toxicity toward both unicellular aquatic organisms and multicellular terrestrial plants.

The applied toxicity assays are methodologically comparable to standardized OECD protocols, although not fully equivalent. The root growth inhibition test using *Sinapis alba* is conceptually consistent with OECD 208, as both evaluate early plant development and root elongation. In contrast, the algal disk diffusion assay represents a simplified and semi-quantitative approach compared to OECD 201, which is based on liquid culture and quantitative evaluation of growth inhibition. Nevertheless, both methods provide relevant information on the biological effects of the tested samples and are suitable for comparative toxicity assessment.

All experiments were performed in triplicate (n = 3). The results are presented as mean values ± standard deviation (SD).

## 3. Results and Discussion

### 3.1. Green Tea Leaf Extract

**Figure 1:**
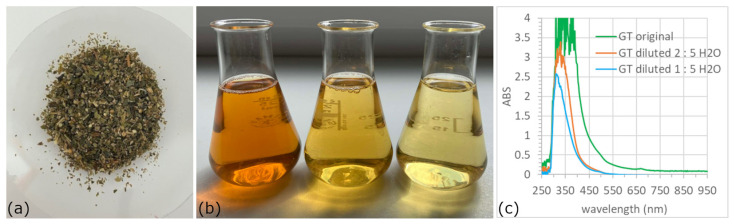
Chopped green tea (GT) leaves (**a**); extracts of green tea leaves: from left to right, original, diluted 2:5, and diluted 1:5 (**b**); UV–Vis spectra of green tea extracts (**c**).

Green tea leaves, Figure 1a represent a well-established biological system for the green synthesis of metal nanoparticles due to their chemically active and complex composition. They are rich in polyphenolic compounds, particularly catechins such as epigallocatechin gallate (EGCG), epicatechin, and epicatechin gallate, which exhibit strong redox properties. In addition, green tea extracts contain flavonoids, amino acids (notably L-theanine), proteins, polysaccharides, and organic acids. These components collectively enable both the reduction of metal ions and the stabilization of the formed nanoparticles [19]. The high density of hydroxyl groups in catechins facilitates electron donation, thereby reducing Au^3+^ ions to metallic Au^0^. At the same time, these molecules adsorb onto the nanoparticle surface, acting as capping agents and preventing aggregation.

The involvement of functional groups in this process has been extensively characterized by Fourier-transform infrared spectroscopy (FTIR) [19,20,21]. Literature FTIR data indicate the presence of absorption bands corresponding to O–H stretching (3200–3500 cm^−1^), C=O stretching of carbonyl or amide groups (1600–1700 cm^−1^), and C–O or C–N vibrations (1000–1400 cm^−1^) [19,20,21]. The well-defined chemical composition and abundance of functional groups make green tea extract a highly effective, reproducible, and environmentally friendly system for the controlled biosynthesis of gold nanoparticles with tunable physicochemical properties.

The dilution of green tea extracts visibly affected their color intensity, Figure 1b. The undiluted extract exhibited a deep yellow color, whereas the 2:5 and 1:5 diluted extracts appeared progressively lighter, indicating a lower concentration of chromophoric compounds. This observation was confirmed by UV–Vis spectroscopy, Figure 1c, which showed a gradual decrease in absorbance intensity with increasing dilution, while the position (λ_max_) of the absorption maximum (ABS_max_) remained nearly unchanged.

The pronounced absorption band observed in the UV region (~270–350 nm) is primarily attributed to polyphenolic compounds in green tea, particularly catechins such as epigallocatechin gallate (EGCG). These compounds contain aromatic rings and conjugated systems, which are responsible for strong electronic transitions (π → π* and n → π*) in this spectral region [20,21]. The high intensity of this band reflects the high concentration of these bioactive molecules, which act as key reducing and stabilizing agents in the synthesis process. Since these compounds are responsible for both the reduction of Au^3+^ ions and the stabilization of the resulting nanoparticles, their decreased concentration may reduce the extract’s reducing capacity and consequently affect the efficiency of AuNP synthesis.

### 3.2. AuNPs Colloids

Gold nanoparticle (AuNP) colloids were synthesized by adjusting the pH of a HAuCl_4_ precursor solution (50 mg·L^−1^). The initial pH of the HAuCl_4_ precursor solution was 1.19, and subsequent samples were prepared by gradual alkalization from 1.19 to 12 by the addition of 10% NaOH solution.

**Figure 2:**
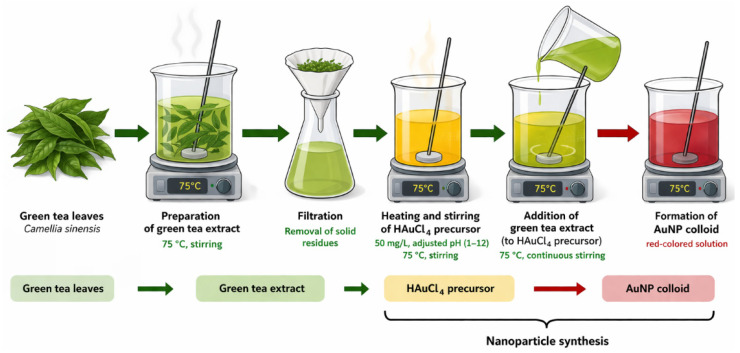
Nanoparticle synthesis process diagram.

Green tea extracts of different concentrations (undiluted, 2:5, and 1:5 dilutions) were subsequently added, and nanoparticle formation was monitored. The scheme of AuNPs colloid preparation is shown in Figure 2. AuNP colloids synthesized under different pH conditions were characterized by UV–Vis spectroscopy, TEM, and biological assays to evaluate the influence of synthesis parameters on nanoparticle properties.

**Figure 3:**
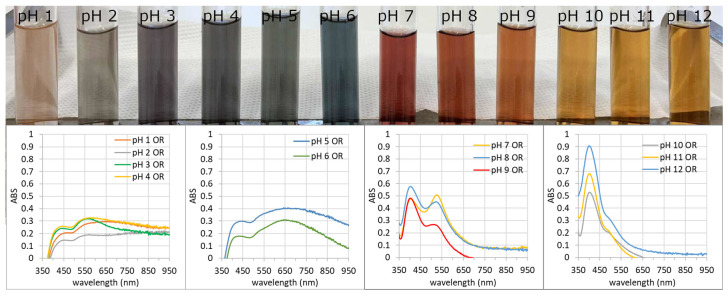
The coloration of solutions and UV–Vis spectra after AuNP synthesis by OR extract.

**Figure 4:**
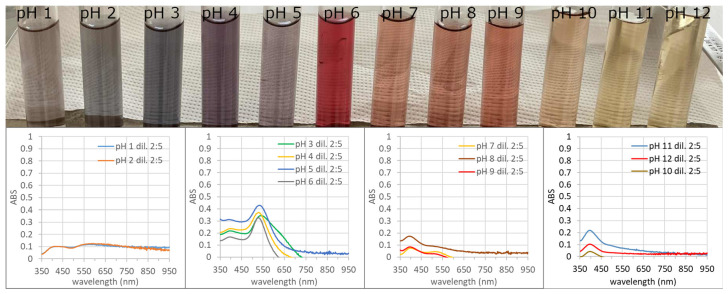
The coloration of solutions and UV–Vis spectra after AuNP synthesis by diluted extract 2:5.

**Figure 5:**
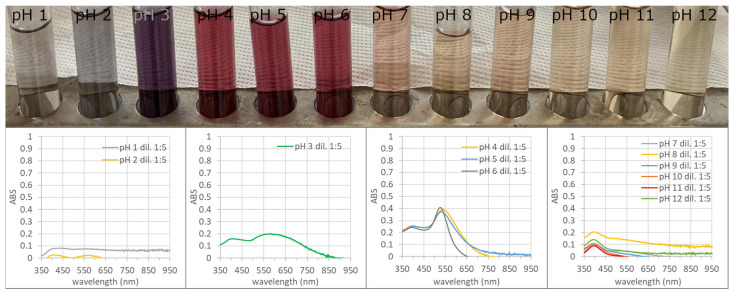
The coloration of solutions and UV–Vis spectra after AuNP synthesis by diluted extract 1:5.

Following the addition, a visible color change occurred within approximately 5 min, indicating AuNP formation, Figure 3, Figure 4 and Figure 5. Depending on pH and extract concentration, the colloids can be classified into four groups: beige, gray, red, and yellow. These differences were reflected in the UV–Vis spectra, which showed substantial variations in absorption maxima, band width, and spectral shape.

For diluted extracts (2:5 and 1:5), nanoparticle formation was strongly suppressed at low pH (1–2) and high pH (≥7), particularly for the 1:5 dilution. The corresponding UV–Vis spectra exhibited low absorbance intensity, indicating insufficient reduction of Au^3+^ ions due to the lower concentration of active biomolecules responsible for reduction and stabilization, Figure 4 and Figure 5.

The strongest effect of extract dilution was observed in the pH range 4–6. Compared with the undiluted extract, dilution resulted in pronounced changes in the colloids’ color and UV–Vis spectra. At pH 4 and 5, the colloids changed from gray to violet, whereas at pH 6, a raspberry-red coloration was obtained. At pH 4, 5, and 6, the undiluted system exhibited a broad absorption profile, and ABS_max_ was localized at λ_max_ ~650 nm. Upon dilution, a blue shift of the absorption maximum, together with a narrowing of the absorption band, was observed. These spectral changes not only indicate modifications in the optical properties of the colloidal system but also suggest differences in other characteristics (size and shape) of the formed nanoparticles.

These findings confirm that pH and extract concentration are key parameters governing the green synthesis of AuNPs and provide an effective means for tuning their optical and morphological properties.

#### 3.2.1. Analysis of Selected Colloids

Since AuNPs are widely utilized in optical applications, the ability to prepare stable colloids with tunable optical properties was considered a key factor in the selection of synthesis conditions. Experimental conditions for further analysis were therefore selected based on the optical properties of the prepared colloids, as the colloids’ color directly reflects the size, shape, and aggregation state of nanoparticles. The selected colloidal conditions included:

- pH 3 and 4 prepared using the 1:5 diluted extract;
- pH 6 prepared using the 2:5 diluted extract;
- pH 6, 9, and 12 prepared using undiluted extract (OR).

**Figure 6:**
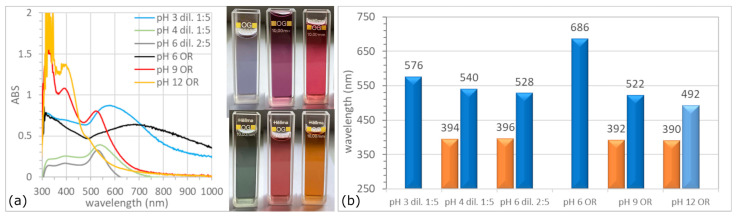
UV–Vis spectra and color of colloids: the first row from left to right—pH 3 (1:5), pH 4 (1:5), pH 6 (2:5); the second row from left to right—pH 6, pH 9, pH 12 (**a**); and histogram of ABS_max_ wavelength of selected AuNP colloids prepared using different extract concentrations and adjusted pH (**b**) (blue—wavelength of ABS_max_ of main peak, yellow—wavelength of ABS_max_ of second peak, light blue—main wavelength of shoulder presented at pH 12).

The corresponding UV–Vis spectra, Figure 6a, show pronounced differences in peak position, number, and spectral width. Normalized UV–Vis spectra of the selected representative samples are provided in the Supplementary Materials (Figure S1) to facilitate comparison of spectral shape and peak position.

Although appropriate blank correction was applied during UV–Vis measurements, absorption bands in the 300–350 nm region were consistently observed (mainly at higher pH). These originate from non-plasmonic contributions, including transformed polyphenols and metal–organic complexes formed during synthesis, as the reaction mixture differs chemically from the reference solution. As a result, the 300–350 nm region may exhibit significant absorbance that is not directly associated with the plasmonic properties of AuNPs. Consequently, the evaluation of nanoparticle formation and comparison of optical properties were focused primarily on wavelengths above 350 nm, where the localized surface plasmon resonance of AuNPs dominates and provides more direct insight into nanoparticle size, morphology, and dispersion.

**Figure 7:**
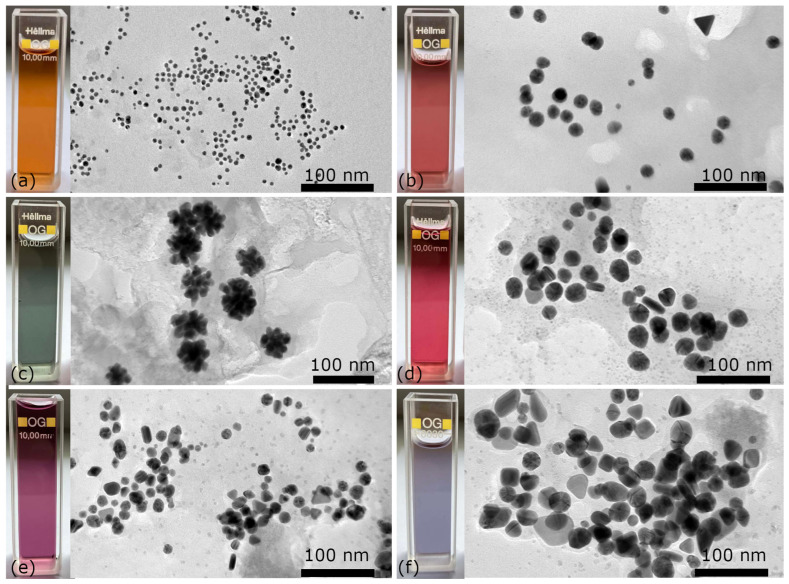
TEM micrographs and related colloids: pH 12 (**a**), pH 9 (**b**), pH 6 (**c**), pH 6 (2:5) (**d**), pH 4 (1:5) (**e**), pH 3 (1:5) (**f**).

At pH 12 (OR), the spectrum is dominated by a narrow peak at ~390 nm with a weak shoulder at ~490 nm, Figure 6a,b, indicating a uniform population of very small, spherical nanoparticles. This is consistent with the yellow coloration and TEM results, Figure 7a, which confirm monodisperse spherical particles with a mean size of 6 ± 1 nm, Table 1. Detailed particle size distribution histograms obtained from TEM analysis for all samples are presented in the Supplementary Materials (Figures S2–S5). These data were used to calculate the mean particle diameter, standard deviation, and other statistical parameters discussed in the manuscript.

In contrast, the spectrum at pH 9 (OR) exhibits multiple features, Figure 6a, including a pronounced SPR band at 528 nm and an additional band near 400 nm, Figure 6b, corresponding to predominantly spherical nanoparticles (18 ± 3 nm) with minor contributions from anisotropic shapes (4%), Table 1, as confirmed by TEM, Figure 7b. Results from other authors confirmed that spherical gold nanoparticles of this size exhibit a red colloidal coloration, which is typically associated with a surface plasmon resonance (SPR) band in the wavelength range of approximately 500–550 nm [16,22], which correlates with our results for samples with pH 9.

A markedly different SPR band is observed at pH 6 (OR), where the spectrum is significantly broadened (ABS_max_ at λ_max_ 686 nm), Figure 6a,b, indicating high polydispersity and anisotropic structures; in this case, green-gray coloration of the colloid was observed. This is consistent with the TEM results showing larger (53 ± 8 nm), irregular, and flower-like nanoparticles, Figure 7c.

Upon dilution (pH 6, (2:5)), the spectrum becomes narrower with a defined mean peak at ~528 nm, Figure 6b, reflecting a shift toward more uniform, predominantly spherical nanoparticles (23 ± 4 nm, 94%), Figure 7d, although minor fractions of rods, polygonal nanoparticles with pentagonal and hexagonal symmetry, and triangular prisms are still present, Table 1. The resulting solution exhibited a raspberry-red coloration.

For diluted systems at lower pH, the spectra further confirm increased heterogeneity. At pH 4 (1:5), a broad absorption band with a maximum at 540 nm, Figure 6a,b indicates a polydisperse system dominated by spherical and quasi-spherical nanoparticles (13 ± 5 nm, 81%), with additional anisotropic shapes such as triangular nanoprisms, nanorods, and faceted polyhedral structures (19%), as was confirmed by TEM, Figure 7e. At pH 3 (1:5), a single broad and red-shifted peak (~576 nm) reflects a highly heterogeneous system with mixed morphologies and larger particles, including spherical and quasi-spherical (23 ± 7 nm, 59%), triangular nanoprisms, nanorods, and faceted polyhedral structures (41%), Figure 7f. Table 1 summarizes the data on the size, shape, and percentage of nanoparticles in colloids.

TEM analysis confirmed the presence of non-spherical nanoparticles, including polyhedral, rod-like, and triangular structures, particularly in samples synthesized at pH 6 (2:5), pH 3 (1:5), and pH 4 (1:5). Although these particles constitute a minority of the population, their presence is important because anisotropic AuNPs exhibit significantly different plasmonic responses compared to spherical nanoparticles. Although present in smaller amounts, these particles influenced the coloration of the colloidal suspensions. For instance, the comparison of the pH 6 (2:5) and pH 3 (1:5) samples (both systems contain spherical nanoparticles with a similar average size of approximately 23 nm) shows that the colloids display markedly different colors and UV–Vis spectra. Since particle size alone cannot explain these differences, the observed optical response is attributed to the presence and proportion of anisotropic nanoparticles in the particle population. Therefore, while spherical nanoparticles dominate the samples, the contribution of less abundant anisotropic structures remains significant for the resulting optical properties.

#### 3.2.2. Composition of Nanoparticles

**Figure 8:**
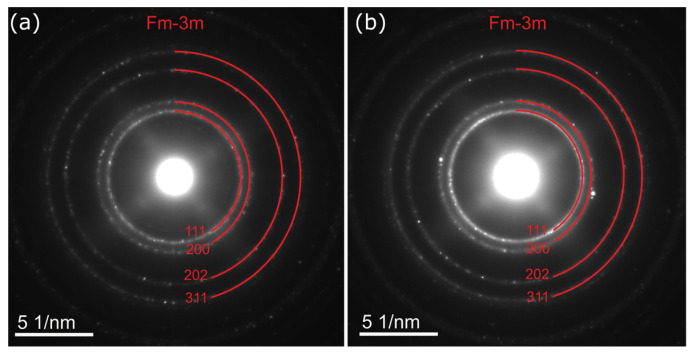
SAED patterns of AuNPs: (**a**) pH 4 (1:5) and (**b**) pH 9 (OR).

Selected area electron diffraction (SAED) analysis was performed for all prepared samples. However, representative patterns are shown for pH 4, Figure 8a, and pH 9, Figure 8b. The pH 4 sample was selected due to the presence of nanoparticles with diverse morphologies, whereas the pH 9 sample represents a system composed predominantly of spherical nanoparticles.

The SAED pattern of AuNPs synthesized at pH 4 confirms the crystalline nature of the nanoparticles. The diffraction pattern exhibits well-defined concentric rings, indicative of a polycrystalline structure composed of randomly oriented nanocrystals. The rings can be indexed to the face-centered cubic (fcc) structure of gold (space group Fm–3m), with reflections corresponding to the (111), (200), (202), and (311) crystallographic planes. The (111) reflection is the most intense, consistent with the preferential stability of this plane in AuNPs.

Similar diffraction features were observed for all analyzed samples, confirming that gold nanoparticles were successfully formed under all experimental conditions. The agreement between SAED, UV–Vis, and TEM analyses further supports the formation of crystalline AuNPs with morphology dependent on synthesis parameters.

In addition to SAED analysis, the presence of gold was confirmed by elemental mapping. Bright-field Scanning Transmission Electron Microscopy (BF-STEM) and High-Angle Annular Dark-Field (HAADF-STEM) imaging were employed to characterize the morphology and composition of the synthesized AuNPs. Although all samples were analyzed, only those prepared at pH 6 and 12 are presented, as they are representative of the detected elemental composition.

**Figure 9:**
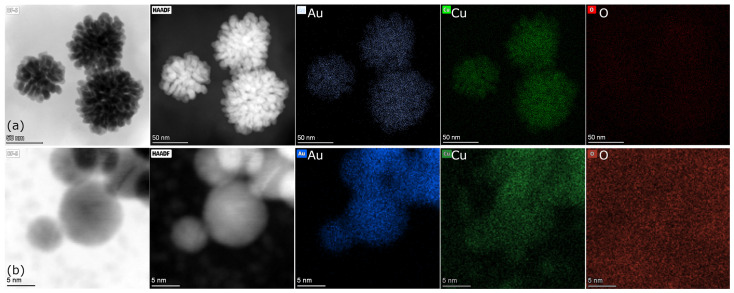
BF-STEM and HAADF-STEM images, and corresponding elemental mapping of AuNPs: pH 6 (**a**) and pH 12 (**b**).

BF-STEM imaging mode, Figure 9a, highlights the overall morphology of the particles, clearly revealing the presence of irregular, flower-like structures. The HAADF images confirm that the observed structures correspond to gold-rich regions, consistent with the elemental mapping results. Elemental mapping of the synthesized AuNPs confirmed the presence of Au, Cl, Cu, and O, Figure 9a,b. The Au signal is localized within the nanoparticle regions, confirming that the observed structures are composed of gold. The presence of chlorine (Cl) can be attributed to residual precursor species originating from HAuCl_4_, such as AuCl^4−^ or partially reduced gold-chloride complexes, which may remain adsorbed on the nanoparticle surface or in the surrounding matrix. This is consistent with incomplete ligand removal during synthesis and indicates that chloride ions can persist as surface-bound species even after nanoparticle formation.

The detected Cu signal originates from the copper TEM grid used as the sample support. The presence of Cu in the elemental maps is not evidence of copper incorporation into the nanoparticles. The apparent overlap between Cu and Au signals is attributed to contributions from the underlying Cu grid and the limited spatial resolution of the EDS mapping technique. The oxygen (O) signal is likely associated with organic compounds from the green tea extract and with possible oxidation products or adsorbed species on the nanoparticle surface. The presence of oxygen peaks in biological AuNPs was also identified by Kalantari et al., who attributed it to organic molecules that probably form the cap on nanoparticles [23].

In addition to Au, Cu, and O, Figure 9b, minor signals of Cl, Na, and Si were also detected in the elemental mapping. The Na signal can be attributed to residual sodium ions originating from NaOH used for pH adjustment, which may remain adsorbed in the sample matrix after drying. The Si signal is most likely associated with trace contamination from sample handling, glassware, dust particles, or background contributions during EDS analysis rather than with the nanoparticles themselves. Since neither Na nor Si is expected to form the core of the synthesized particles, these signals should be regarded as secondary contributions from the preparation and measurement environment.

#### 3.2.3. Toxicity of AuNPs

The potential toxicity of the synthesized gold nanoparticles was evaluated using two biological model systems representing different levels of biological complexity. The first model involved the green microalga *Ch. kessleri*, which is commonly used for assessing nanoparticle effects in aquatic environments. The second model consisted of higher plant assays based on *Sinapis alba* root growth inhibition. This combined approach allows for a comprehensive evaluation of nanoparticle toxicity, considering both unicellular and multicellular organisms, as well as different exposure pathways.

**Figure 10:**
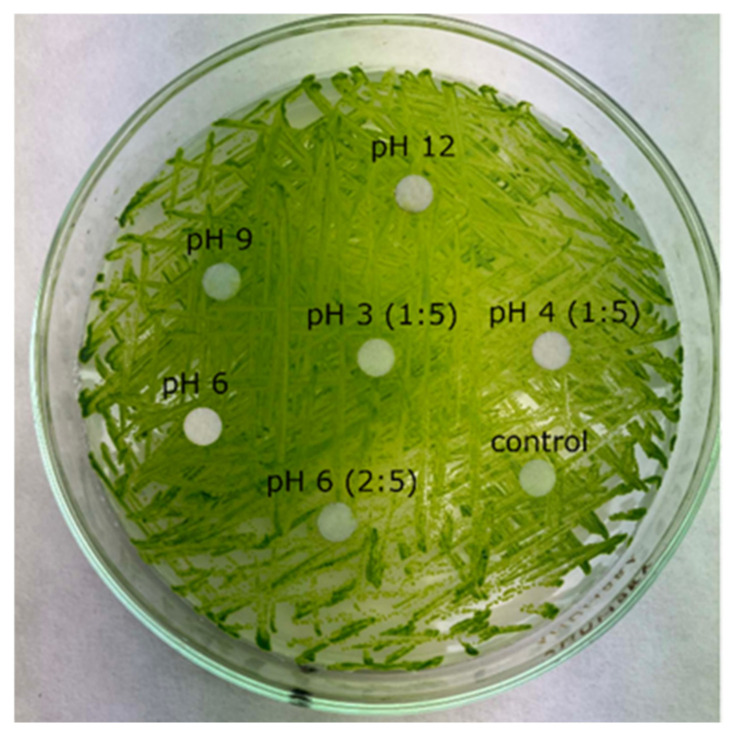
Disk diffusion test on algae *Ch. kessleri*.

The toxicity assessment performed using *Ch. kessleri* demonstrated that none of the synthesized AuNP samples and controls (H_2_O) induced growth inhibition, regardless of pH conditions or nanoparticle morphology, Figure 10. No inhibitory zones or reduction in algal proliferation were observed, indicating that the prepared gold nanoparticles exhibit negligible toxicity under the tested conditions.

These findings are consistent with previously reported studies highlighting the low toxicity of gold nanoparticles in aquatic and photosynthetic systems. Behra et al. [24] showed that AuNPs exhibit limited toxicity toward Chlamydomonas reinhardtii, with observed effects strongly dependent on colloidal stability and particle concentration rather than the presence of gold itself. Similarly, Ostroumov et al. [25] reported that gold nanoparticles do not significantly inhibit plant growth in aquatic systems, supporting their relatively inert behavior. The work of Glenn et al. [26] further demonstrated that interactions of AuNPs with aquatic plants are size- and species-dependent, with no universal toxic effect observed. In addition, Contini et al. [27] emphasized that interactions of AuNPs with biological systems are governed primarily by particle size and surface properties, which influence their interaction with biological membranes rather than inducing direct toxicity.

Taken together, these studies support the conclusion that gold nanoparticles, particularly those synthesized via green methods and stabilized by biomolecules, exhibit low intrinsic toxicity. The absence of inhibitory effects observed in this study is therefore in good agreement with the literature and suggests that the prepared AuNPs are biocompatible within the tested experimental conditions.

In contrast to the algal toxicity test, inhibition of root growth was observed in higher plants (*Sinapis alba*), indicating increased sensitivity of plant systems to the tested samples. This behavior is consistent with literature reports showing that gold nanoparticles can affect plant growth, particularly root development, depending on particle size, concentration, and plant species.

**Figure 11:**
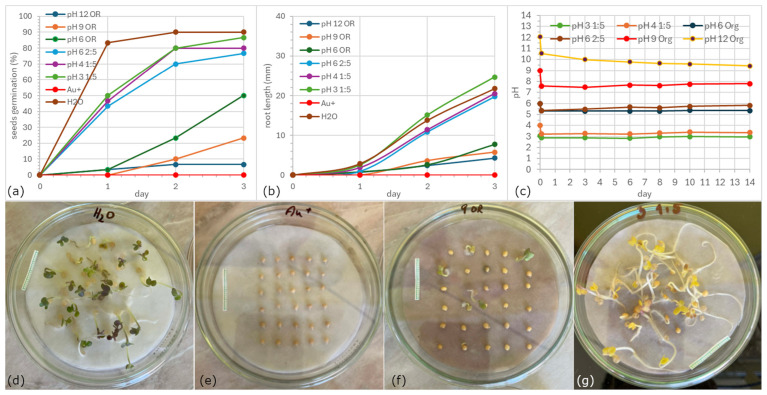
The percentage of germinated seeds (**a**), root length over time (**b**), and pH of tested colloids (**c**). Photographs of seed germination and root development on the 3rd day of exposure: positive control H_2_O (**d**), negative control Au^+^ (**e**), sample pH 9 (**f**), and pH 3 (**g**). Error estimates (mean ± SD, n = 3) are provided in Tables S1 and S2 in the Supplementary Materials.

The obtained results indicate that smaller AuNPs exert a stronger inhibitory effect on root germination and elongation. AuNPs with sizes ranging from approximately 5 to 20 nm (samples prepared at pH 12 and 9) exhibited low germination rates, not exceeding 30%, Figure 11a,f. These samples also showed the shortest root lengths, reaching only up to ~8 mm on the third day of the experiment, Figure 11b,g.

In contrast, nanoparticles prepared at lower pH values, characterized by mixed morphologies and average sizes more than 25 nm, exhibited significantly lower phytotoxicity. Their germination rates and root growth were considerably closer to the positive control (H_2_O), where approximately 90% germination was achieved, and green leaves were already visible by the third day of cultivation, Figure 11d. Complete inhibition of root growth was observed only for the negative control containing ionic gold solution (Au^3+^), Figure 11a,e. This observation confirmed that free ionic gold species were not significantly present in the prepared colloidal systems and that the reduction of Au^3+^ to Au^0^ during synthesis was highly efficient. Detailed germination and root elongation results, including mean values and standard deviations obtained from three independent experiments, are provided in the Supplementary Materials (Tables S1 and S2). The low variability between replicates confirms the good reproducibility of the bioassays and supports the observed differences among the tested samples. Complete inhibition of root growth was observed for the Au^+^ control, whereas AuNP suspensions synthesized at pH 3 (1:5) and pH 4 (1:5) promoted root development comparable to, or even slightly exceeding, that of the water control.

The temporal evolution of pH values over 10 days is shown in Figure 11c. No significant pH changes were observed after synthesis, confirming both the stability of the colloidal systems and the rapid establishment of equilibrium following nanoparticle formation.

However, the observed phytotoxic effects cannot be attributed solely to nanoparticle size. Previous studies have shown that the toxicity of gold-based systems may also be influenced by environmental conditions, including pH. Nevertheless, in the present study, inhibitory effects were also observed for the sample prepared at pH 6, where highly branched flower-like nanoparticles were formed. Since this pH range itself is generally suitable for root growth, the results suggest that nanoparticle morphology also plays an important role in determining biological response. It should be noted that residual phytochemicals originating from the green tea extract may also contribute to the observed biological responses. Green tea extracts are rich in catechins and polyphenolic compounds with well-documented antioxidant properties and generally low toxicity. However, the same extract was used for all synthesis conditions, whereas the toxicity varied considerably among the individual AuNP samples. Furthermore, the Au^+^ control completely inhibited seed germination and root growth, while the synthesized AuNPs exhibited markedly different biological effects depending on their size and morphology. These findings indicate that residual extract components or dissolved gold species were not the primary factors responsible for the observed phytotoxicity. Instead, nanoparticle characteristics, particularly size and shape, appear to play a dominant role in determining their interaction with higher plants.

The synthesized AuNPs exhibited negligible toxicity toward algae; their effects on higher plants were more complex, clearly demonstrating that nanoparticle size and morphology significantly influence plant viability and development.

## 4. Conclusions

In conclusion, this study demonstrates that both pH and extract concentration are critical parameters governing the biological synthesis of gold nanoparticles using green tea extract. The results clearly show that pH strongly influences the reduction efficiency of Au^3+^ ions, nanoparticle nucleation and growth, and the resulting optical properties, size distribution, and morphology.

While strongly acidic and highly alkaline conditions were generally unfavorable for controlled nanoparticle formation, particularly in diluted systems, the intermediate pH range (3–6) provided the most suitable conditions for AuNP synthesis. Nevertheless, even under these conditions, the nanoparticles exhibited a certain degree of polydispersity and morphological diversity, including spherical, anisotropic, and irregular structures.

A broad range of nanoparticle morphologies was obtained depending on the synthesis conditions. The prepared systems included spherical nanoparticles with average diameters of approximately 6 nm and 20 nm, as well as flower-like structures with average sizes around 50 nm. In addition, mixed populations of spherical and anisotropic nanoparticles were associated with the formation of raspberry-red, violet, and blue colloids. These findings demonstrate that the optical response of the synthesized AuNPs can be effectively tuned through variation of pH and extract concentration.

However, further optimization is required to achieve greater control over nanoparticle morphology, particularly for the selective preparation of triangular nanoprisms or nanorods. Future work will therefore focus on refining synthesis parameters to better control anisotropic growth and to further investigate the relationship between nanoparticle morphology and optical properties.

Toxicity assessment revealed no inhibitory effects on the growth of *Chlorella kessleri*, whereas root growth inhibition was observed in *Sinapis alba*, indicating a higher sensitivity of multicellular plant systems. The results further suggest that nanoparticle characteristics, particularly size and morphology, play an important role in determining their biological effects.

## Figures and Tables

**Table 1:** Size, shape, percentage of nanoparticles in colloids, and number of measured particles.

	Nanoparticles Shape										Number of Particles
	Spheres/ Quasi-Spheres		Triangular Nano Prisms		Polyhedral Structures		Nanorods Length: Width		Flower-like		
	(nm)	(%)	(nm)	(%)	(nm)	(%)	(nm)	(%)	(nm)	(%)	
pH 12	6	100	-	-	-	-	-	-	-	-	418
pH 9	18	96	39	4	-	-	-	-	-	-	151
pH 6	-	-	-	-	-	-	-	-	53	100	80
pH 6 2:5	23	94	27	3	20	1	31:13	2	-	-	205
pH 4 1:5	13	81	17	8	16	9	21:11	2	-	-	405
pH 3 1:5	23	69	25	15	24	15	39:11	1	-	-	195

## Data Availability

The original contributions presented in this study are included in the article. Further inquiries can be directed to the corresponding author.

## Supplementary Materials

The following supporting information can be downloaded at: https://www.mdpi.com/article/10.3390/ma19132780/s1, File: SuplemMaterials.pdf. Figure S1: Normalized UV–Vis spectra of selected AuNP samples synthesized under different pH conditions and extract concentrations. Figure S2: Histogram of nanoparticle size distribution for samples pH 6, 9, and 12. Figure S3: Histogram of nanoparticle size distribution for samples pH 6 (2:5). Figure S4: Histogram of nanoparticle size distribution for samples pH 4 (1:5). Figure S5: Histogram of nanoparticle size distribution for samples pH 3 (1:5). Table S1: Germination assay results obtained from three independent experiments (n = 3). Values are presented as the mean ± standard deviation (SD) of the number of germinated seeds. Table S2: Root length assay results obtained from three independent experiments (n = 3). Values are presented as mean ± SD (mm).
