# Supplementary material for: Study of pH Role in the Green Synthesis of Gold Nanoparticles: From Reduction to Size and Shape Control
Source: Materials (Basel). 2026 Jun 30;19(13):2780. doi: 10.3390/ma19132780 (PMC13362575; doi:10.3390/ma19132780)
Supplement: Supplementary file 1 [file materials-19-02780-s001.zip › materials-4367939-supplementary.pdf]

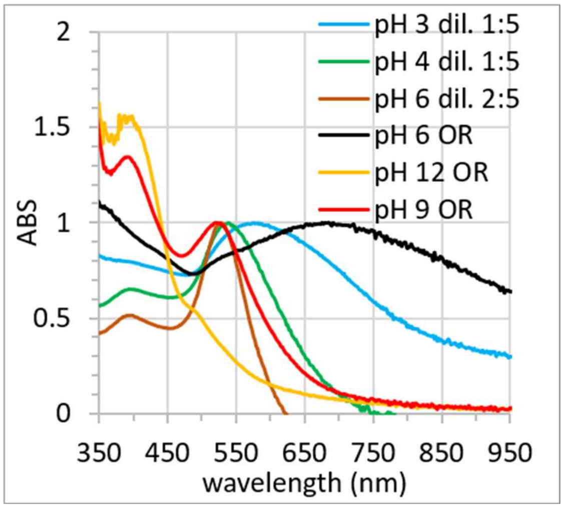

Figure S1 Normalized UV–Vis spectra of selected AuNP samples synthesized under different pH conditions and extract concentrations.

Normalization was performed to facilitate comparison of spectral shape, peak position, and band broadening independently of absorbance intensity. The spectra demonstrate substantial differences in optical response among the samples, reflecting variations in nanoparticle size distribution and morphology. In particular, the broad absorption bands observed for the pH 6 OR and pH 3 (1:5) samples indicate the presence of more heterogeneous nanoparticle populations, whereas the narrower bands recorded for the pH 9 OR and pH 12 OR samples suggest a more uniform particle distribution.

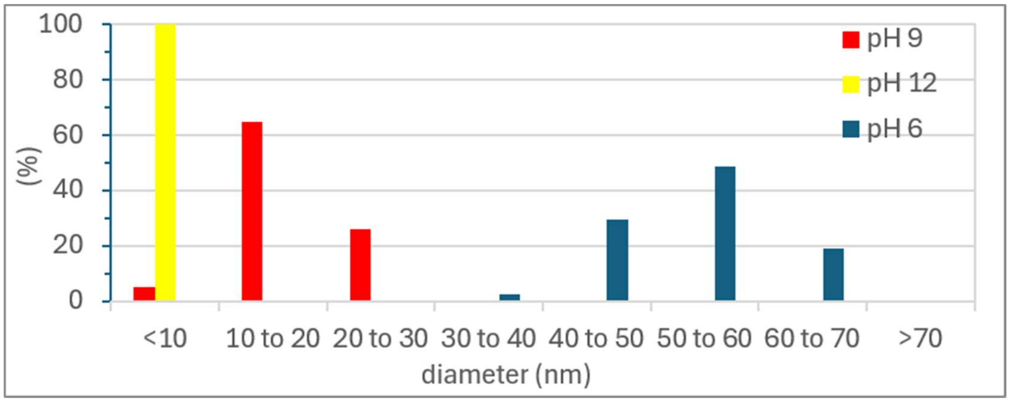

Figure S2 Histogram of nanoparticle size distribution for samples pH 6, 9, and 12

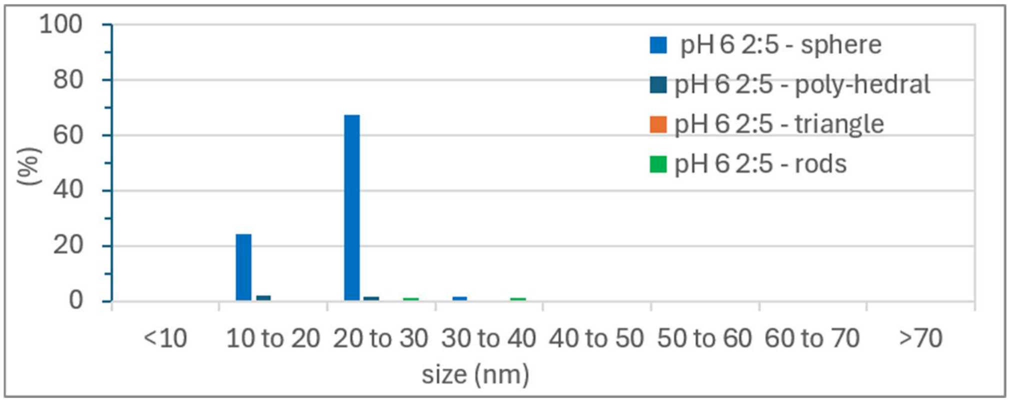

Figure S3 Histogram of nanoparticle size distribution for samples pH 6 (2:5)

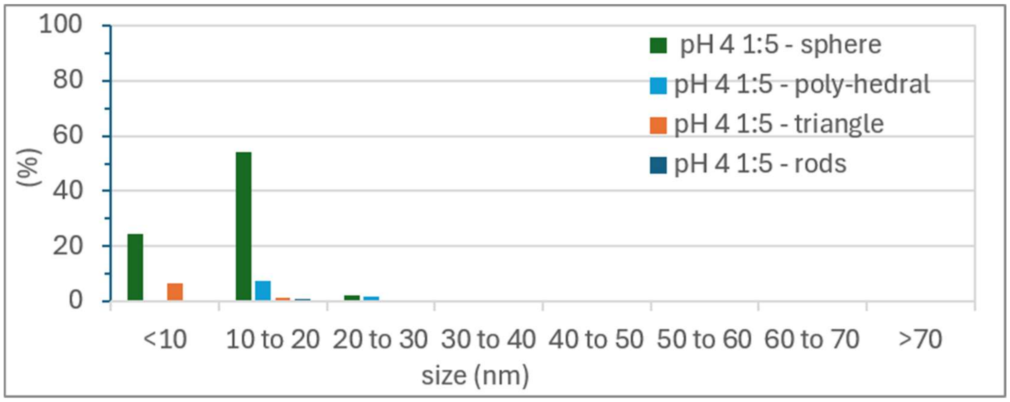

Figure S4 Histogram of nanoparticle size distribution for samples pH 4 (1:5)

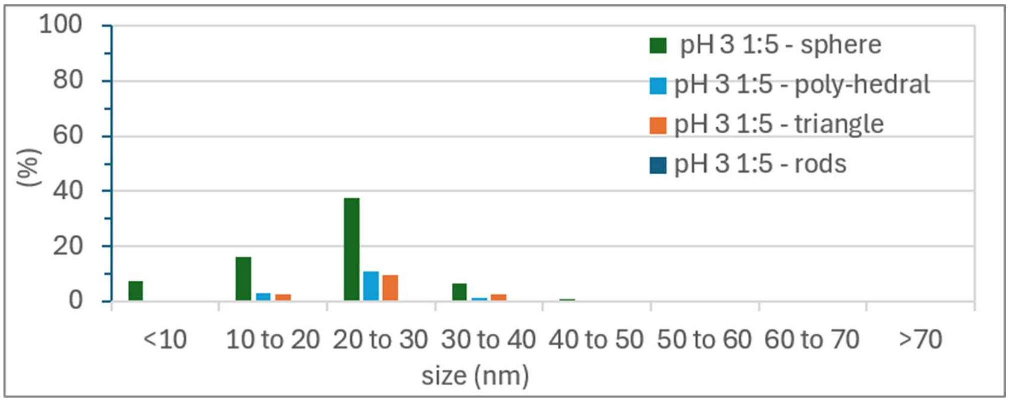

Figure S5 Histogram of nanoparticle size distribution for samples pH 3 (1:5)

Table S1. Germination assay results obtained from three independent experiments (n = 3). Values are presented as the mean ± standard deviation (SD) of the number of germinated seeds.

Sample	Day 1	Day 2	Day 3
pH 12 OR	1.00 ± 1.00	2.00 ± 0.00	2.67 ± 1.15
pH 9 OR	0.33 ± 0.58	3.33 ± 0.58	6.67 ± 0.58
pH 6 OR	1.33 ± 0.58	7.00 ± 1.00	15.00 ± 1.00
pH 6 (2:5)	13.33 ± 0.58	20.67 ± 0.58	23.00 ± 1.00
pH 4 (1:5)	13.67 ± 1.53	24.00 ± 1.00	24.33 ± 0.58
pH 3 (1:5)	15.00 ± 1.00	24.00 ± 1.00	26.00 ± 0.00
Au^+	0.00 ± 0.00	0.00 ± 0.00	0.00 ± 0.00
H_2O (control)	25.00 ± 1.00	26.00 ± 1.00	27.33 ± 0.58

The germination assay was performed in triplicate using mustard seeds exposed to AuNPs suspensions synthesized under different pH conditions and extract concentrations. The table summarizes the mean number of germinated seeds and the corresponding standard deviations obtained during the three-day observation period. The relatively low SD values indicate good reproducibility of the experimental procedure.

Table S2. Root length assay results obtained from three independent experiments (n = 3). Values are presented as mean ± SD (mm).

Sample	Day 1	Day 2	Day 3
pH 12 OR	0.70 ± 0.10	2.47 ± 0.12	4.50 ± 0.44
pH 9 OR	0.00 ± 0.00	3.70 ± 0.56	5.67 ± 0.25
pH 6 OR	0.80 ± 0.20	2.63 ± 0.25	7.67 ± 0.15
pH 6 (2:5)	0.87 ± 0.15	11.27 ± 0.64	19.53 ± 0.31
pH 4 (1:5)	1.87 ± 0.35	11.07 ± 1.17	20.17 ± 2.02
pH 3 (1:5)	2.63 ± 0.15	15.10 ± 0.50	24.87 ± 0.15
Au^+	0.00 ± 0.00	0.00 ± 0.00	0.00 ± 0.00
H_2O (control)	2.83 ± 0.21	13.93 ± 0.53	21.57 ± 0.55

Table S2 summarizes the root elongation assay performed in triplicate. Values are expressed as mean root length ± standard deviation (SD). The results demonstrate substantial differences in root growth among the tested AuNPs suspensions. The ionic gold solution (Au^+) completely inhibited root development, whereas the AuNPs samples synthesized at pH 3 (1:5) and pH 4 (1:5) exhibited root lengths comparable to or exceeding those observed for the water control. The low standard deviations indicate good reproducibility of the measurements.
